# Effects of Isokinetic Training on Trunk Muscle Fitness and Body Composition in World-Class Canoe Sprinters

**DOI:** 10.3389/fphys.2019.00021

**Published:** 2019-01-28

**Authors:** Fridolin Zinke, Torsten Warnke, Martijn Gäbler, Urs Granacher

**Affiliations:** ^1^Division of Training and Movement Sciences, Research Focus Cognition Sciences, University of Potsdam, Potsdam, Germany; ^2^Institute for Applied Training Science, Leipzig, Germany; ^3^Center for Human Movement Sciences, University Medical Center Groningen, University of Groningen, Groningen, Netherlands

**Keywords:** peak torque, canoe racing, core strength, sport-specific performance, elite athletes

## Abstract

In canoe sprint, the trunk muscles play an important role in stabilizing the body in an unstable environment (boat) and in generating forces that are transmitted through the shoulders and arms to the paddle for propulsion of the boat. Isokinetic training is well suited for sports in which propulsion is generated through water resistance due to similarities in the resistive mode. Thus, the purpose of this study was to determine the effects of isokinetic training in addition to regular sport-specific training on trunk muscular fitness and body composition in world-class canoeists and to evaluate associations between trunk muscular fitness and canoe-specific performance. Nine world-class canoeists (age: 25.6 ± 3.3 years; three females; four world champions; three Olympic gold medalists) participated in an 8-week progressive isokinetic training with a 6-week block “muscle hypertrophy” and a 2-week block “muscle power.” Pre- and post-tests included the assessment of peak isokinetic torque at different velocities in concentric (30 and 140°s^-1^) and eccentric (30 and 90°s^-1^) mode, trunk muscle endurance, and body composition (e.g., body fat, segmental lean mass). Additionally, peak paddle force was assessed in the flume at a water current of 3.4 m/s. Significant pre-to-post increases were found for peak torque of the trunk rotators at 30°s^-1^ (*p* = 0.047; *d* = 0.4) and 140°s^-1^ (*p* = 0.014; *d* = 0.7) in concentric mode. No significant pre-to-post changes were detected for eccentric trunk rotator torque, trunk muscle endurance, and body composition (*p* > 0.148). Significant medium-to-large correlations were observed between concentric trunk rotator torque but not trunk muscle endurance and peak paddle force, irrespective of the isokinetic movement velocity (all *r* ≥ 0.886; *p* ≤ 0.008). Isokinetic trunk rotator training is effective in improving concentric trunk rotator strength in world-class canoe sprinters. It is recommended to progressively increase angular velocity from 30°s^-1^ to 140°s^-1^ over the course of the training period.

## Introduction

Canoe sprint is a sport that demands high levels of endurance and muscular fitness to compete at the international level (Tesch, 1983; Fry and Morton, 1991; Borges et al., 2015). The specific metabolic contributions depend on the race distance with primarily anaerobic energy supply during race distances up to 250 m and predominantly aerobic supply during race distances ≥500 m. Critical 1performance determinants of the pull-through phase of the kayak-stroke during short distance racing (e.g., short sprint) are upper limb and trunk muscle strength and power. In race distances ≥500 m, muscular endurance of upper limb and trunk muscles constitute important performance determinants (Trevithick et al., 2007; Uali et al., 2012). Trunk muscle strength and stability are particularly needed in canoe sprint because of the athlete’s unstable position in a rather narrow boat. According to Bergmark (1989), the local trunk muscles (e.g., m. multifidii) stabilize the trunk and spinal column whereas the global muscles (e.g., mm. rectus and obliqui abdominis) are responsible for the transfer of forces and torques from the trunk to the upper limbs, thereby creating propulsion. In addition to the aforementioned trunk muscles, upper limb muscles (e.g., mm. triceps brachii, biceps brachii) contribute to boat propulsion as well (Fry and Morton, 1991; Akca and Muniroglu, 2008; McKean and Burkett, 2010).

Given these canoe-specific findings on the role of trunk muscle strength and stability for performance (Mann and Kearney, 1980), it is somewhat surprising that these results were not supported by findings from a recent systematic literature review and meta-analysis on the general role of trunk muscle strength for physical fitness and athletic performance in healthy trained individuals of different sporting background (e.g., athletics, swimming) (Prieske et al., 2016). Based on findings from 15 correlation studies, Prieske et al. (2016) observed only small-sized relationships between measures of trunk muscle strength and physical performance. In addition, the results of 16 intervention studies indicated only small-to-medium-sized effects of core strength training compared with no training or regular training on proxies of physical performance. Of note, Prieske et al. (2016) discussed a major limitation of their findings and questioned the external validity of the applied trunk muscle strength tests. Most included studies measured trunk muscle strength by means of a trunk muscle endurance test using an isometric plank test. Prieske et al. (2016) postulated that these tests do not appropriately evaluate maximal force production capacities in dynamic sport-specific activities.

In contrast to the findings of Prieske et al. (2016), original work from Fry and Morton (1991) indicates significant and medium-sized correlations (*r* = 0.46–0.69) between peak isokinetic trunk rotation torque during simulated paddle strokes and race time in sprint and endurance race distances in internationally ranked male canoe-athletes. These authors further reported that peak isokinetic torque of the trunk rotators during simulated paddle strokes represents a marker that discriminates between canoeists of high versus low performance levels (i.e., selected vs. non-selected members of a state representative-team) (Fry and Morton, 1991). Based on these findings, strength training in general and trunk muscle training (i.e., core strength training) in particular appear to be promising means for canoe athletes to gain access to not yet recruited physiological reserves of the trunk muscles that are needed for boat propulsion.

McKean and Burkett (2014) examined the effects of a 3-year dry-land strength training program with three to four sessions per week on one-repetition maximum [1-RM] (i.e., bench press, pull up) and race performance time (200, 500, and 1,000 m) in international level elite male and female kayakers. The training protocol included four to six sets with six to eight repetitions at 75–85% of the 1-RM. After the 3-year intervention, the authors observed significant increases in 1-RM bench press and pull up performances in the range of 2.3–13.0% and in all race performance times (200 m: 3.1–9.2%; 500 m: 2.5–7.7%; 1,000 m: 3.1–5.0%) (McKean and Burkett, 2014).

In accordance with the principle of training specificity, strength training stimuli are most effective when training characteristics closely resemble the demands of a specific sport. In this context, Behm and Sale (1993) reported the largest strength gains for movement velocities that were exercised during training. In canoeing, the specific characteristics of the water have to be taken into consideration when designing strength training interventions. Although the movement of the paddle in the water is not exclusively isokinetic, there are marked similarities between the resistive mode in the water and those on the isokinetic dynamometer. Torque-time curves during isokinetic movements have a similar profile as force-time curves during paddling. A further characteristic of isokinetic training compared to other forms of on-land training is the possibility to exert maximal torque throughout the whole range of motion. This unique characteristic of isokinetic training offers an opportunity to provide a sport-specific training stimulus on land that cannot be achieved by other types of strength training (Ostering, 1988). Finally, isokinetic training allows the athlete to exert maximal torque during eccentric muscle contraction over the full range of motion which is currently not feasible with other types of strength training. According to A. V. Hill’s force-velocity relations, higher forces/torques can be achieved during eccentric compared with isometric and concentric muscle actions. As such, (isokinetic) eccentric training potentially increases maximum strength/peak torque and muscle mass more than concentric training (Roig et al., 2009). Even though isokinetic strength training appears to be a promising candidate to enhance performance in elite canoeists, there are currently no studies available that examined the effects of isokinetic trunk rotator training on trunk muscle strength, -mass and -endurance in world-class canoe sprinters.

Thus, the main objective of this study was to evaluate the effects of isokinetic trunk rotator training in combination with canoe-specific and athletic training on peak torque of the trunk rotators, trunk muscle endurance, and body composition in world-class canoe sprinters. With reference to the relevant literature (McKean and Burkett, 2014), we hypothesized that peak torque of the trunk rotators and lean body mass increase over the course of the 8-week isokinetic training period. A secondary objective was to analyze associations between trunk muscle fitness (i.e., peak trunk rotator torque, trunk muscle endurance) and canoe-specific performance (i.e., paddle force). In accordance with previous studies (Fry and Morton, 1991), we hypothesize that medium-to-large correlations exist between trunk muscle fitness and canoe-specific performance.

## Materials and Methods

### Participants

Nine world-class canoe sprinters (sport: seven kayak/two canoe; sex: three females/six males) who compete on an international/Olympic level (four world champions; three Olympic gold medalists) volunteered to participate in this study (Table 1). All athletes were free of any musculoskeletal, neurological, or orthopedic disorders prior to and during the study. All participants gave written informed consent in accordance with the latest version of the Declaration of Helsinki and all experimental procedures were approved by the local ethics committee (University of Potsdam).

**Table 1:** Characteristics of the study sample (means and standard deviations).

Age (years)	25.6 ± 3.3
Height (cm)	187.5 ± 8.1
Body mass (kg)	82.9 ± 9.3
Fat mass (%)	8.8 ± 4.6
Skeletal muscle mass (kg)	43.6 ± 6.5
Body mass index (kg/m^2^)	23.5 ± 0.8

### Protocol

A single group intervention study was designed to examine the effects of isokinetic training in addition to regular training on muscle fitness and body composition in world-class canoeists. In this context, muscular fitness is used as an umbrella term for muscular strength, local muscular endurance and muscular power (Granacher et al., 2016). Additionally, associations between isokinetic torque and canoe specific performance were computed. The study sample comprised world-class athletes. A limitation of such an expert population is the small sample. Given that the number of world-class athletes in one sporting nation is limited, we were not able to implement an active control in our study design.

Study participants conducted an 8-week isokinetic strength training for the trunk rotator muscles during the pre-season (October–December 2017). Before and after the intervention, all athletes were tested for their peak isokinetic trunk rotator torque and trunk muscle endurance using a plank test. Six athletes were tested for their body composition before and after the intervention. Athletes started with the test for body composition in the morning of the testing day. After a 2-h breakfast break, participants continued with a standardized isokinetic warm-up program for the trunk muscles (i.e., two submaximal sets of eight repetitions at 30°s^-1^ and two submaximal sets of eight repetitions at 140°s^-1^) that was followed by the assessment of maximal trunk muscle strength on an isokinetic dynamometer. Thereafter, participants conducted the trunk muscle endurance test. All tests were performed at the same time of day during pre and post assessment. The graded canoe specific test in the flume was performed by seven athletes during the first week of the intervention. On this specific day, no isokinetic testing or training was performed. We were not able to repeat this test because athletes were on their way to a training camp.

### Body Composition

Body mass and percent body fat were quantified by means of a bioimpedance analysis system (Inbody 720, Biospace, Seoul, South Korea). All measures were taken in consideration of current standards (e.g., no intake of food up to 12 h prior the testing/no caffeine before the measurement/no alcohol the day before measurement). Pre- and post-tests were conducted in the morning. The test can be classified as valid with intraclass correlation coefficients (ICCs) between 0.97 and 0.99 for whole body lean mass and fat mass and ICCs between 0.91 and 0.70 for segmental lean muscle mass quantifications (Ling et al., 2011). The segmental analysis allows to distinguish between the left and right arm/leg and trunk.

### Maximum Isokinetic Strength

**FIGURE 1:**
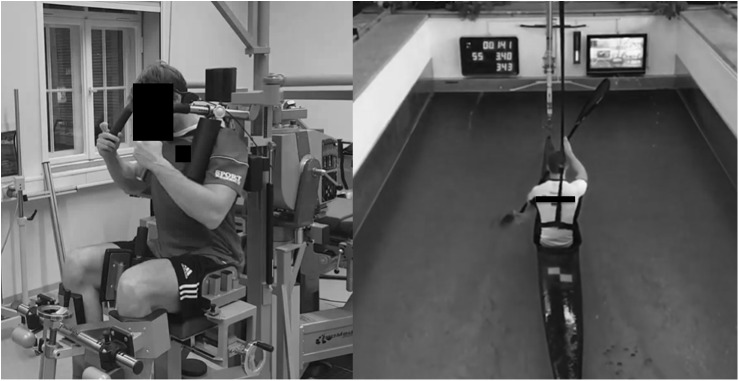
**(A)** Shows an athlete during testing or training on the isokinetic dynamometer. **(B)** Illustrates an athlete during the canoe-specific performance test in the flume at a velocity of 3.4 ms^-1^. Consent for publication of both images was obtained.

Peak isokinetic torque of the trunk rotators was measured using an isokinetic dynamometer (Isomed2000, D&R Ferstl GmbH, Hemau, Germany). After individual adjustments, participants were seated in a rigid chair of the isokinetic device, with the knee and ankle angle adjusted at 90° and the hip angle adjusted at 80°. The upper body was slightly bent forward to resemble the position in the boat. Straps/pads fixed the knees, hip, trunk, and shoulders in place (Figure 1A). The individually recorded position on the dynamometer was maintained throughout all testing and training procedures. Tests of peak isokinetic torque were performed from right rotation (37°) to left rotation (37°) which results in a total range of motion of 74° (Bjerkefors et al., 2018).

After a warm-up that included two submaximal sets of eight repetitions at 30°s^-1^ and two submaximal sets of eight repetitions at 140°s^-1^, isokinetic testing was conducted. Pre- and post-tests comprised the assessment of peak isokinetic torque at different velocities in concentric (30°s^-1^ and 140°s^-1^) and eccentric (30°s^-1^ and 90°s^-1^) mode. One-minute rest separated the trials. The best trial of the three repetitions with the highest peak torque output was used for further analysis. A similar protocol on the same type of dynamometer showed ICCs ranging between 0.87 and 0.91 (Roth et al., 2017).

### Trunk Muscle Endurance

The ventral Bourban test was used to assess trunk muscle endurance. Participants were in prone plank position on their elbows and toes. While in the plank position, the lower horizontal reference rod of the alignment device was attached to the participant’s lower back at the level of the iliac crests and was then fixed in this position. After visual inspection of the participant’s starting position, athletes were asked to lift their feet alternately for 2–5 cm according to the beat of a metronome (i.e., 1 s per foot). Before testing, participants were instructed to constantly remain in contact with the horizontal reference rod for as long as possible. The test was terminated when participants failed to remain in contact with the reference rod for the third time or at exhaustion. One test trial was performed. Test duration was measured to the nearest second using a hand-held stop watch (Lesinski et al., 2017). The test can be classified as reliable with a coefficient of variation of 14.1% (Tschopp et al., 2001).

### Canoe-Specific Performance

Seven of the included athletes performed a canoe-specific performance test in the flume under standardized laboratory conditions (i.e., constant air- and water temperature) (Figure 1B). The test comprised a maximum number of seven stages with a 1,000 m stage length. Water velocity was increased by 0.2 ms^-1^ for each stage. The starting velocity was either 3.2 or 3.4 ms^-1^ based on individual test results from the previous seasons. The rest period between every stage was 6 min. During rest, athletes stayed in the boat that was fixed and stabilized using the reference rod of the alignment device. During the test, the boat position in the flume was monitored using an online feedback system (traffic light) that was directly installed in front of the athlete. If the boat moved out of this position for more than one second, an acoustic warning signal turned on. The test was terminated after three warnings. Peak paddle force was assessed at a sampling frequency of 200 Hz using an instrumented paddle with a strain gauge (UMKS, FES; Berlin, Germany) at a water velocity of 3.4 ms^-1^. Any higher or lower water velocity would result in fewer participants because some athletes aborted at 3.6 ms^-1^. The strain gauge system was calibrated for the individual grip position of the athlete.

### Isokinetic Training

Testing and training were carried out on the same isokinetic trunk rotation dynamometer (Isomed2000, D&R Ferstl GmbH, Hemau, Germany). The intervention was implemented during the pre-season and was performed in combination with canoe specific and other athletic training (e.g., free weight strength training). All training sessions were carried out in the afternoon following an athletic training session. Every isokinetic training session lasted approximately 20 min.

Progression during isokinetic training was realized using time under tension (TUT). For this purpose, the training frequency increased from two sessions per week during the first 2 weeks to three sessions per week from week 3 to week 8. Additionally, movement velocity increased from week 1 (30°s^-1^) until week 8 (140°s^-1^). The increase in TUT from weeks 1 to 3 was followed by a detraining in week 4 to allow better adaptation. In other words, a 3:1 loading paradigm was chosen during the hypertrophy block (weeks 1–6). In the power block (weeks 7–8), TUT continuously increased over both weeks. The average increase in TUT was 26% during the hypertrophy block and 21% during the power block. The first 6 weeks of isokinetic training focused on muscle hypertrophy. Training was conducted in concentric and eccentric mode using the same load parameters. During the hypertrophy block, slow movement velocities (30–60°s^-1^) were realized. The last 2 weeks of training focused on muscle power. Only concentric muscle actions at fast movement velocities (100–140°s^-1^) were performed during this block (weeks 7–8). Pilot studies indicated that eccentric actions were not feasible at high movement velocities.

Additionally, the Short Recovery and Stress Scale (SRSS) was applied before the first training session of every single training week to test overall recovery and stress of the participating athletes. The scale comprises eight items on a 7-point Likert scale. Four items target recovery-related issues and four items stress-related issues (Kölling et al., 2015). If the SRSS indicated high stress and little recovery, we refrained from progressing the training load from 1 week to the other. If the report revealed normal stress levels, the previously described loading paradigm was applied. The movement velocity as well as all other load parameters (e.g., sets, repetitions, and rest between sets) are illustrated in Table 2.

**Table 2:** Training protocol of the 8-week isokinetic training intervention.

	Hypertrophy						Power	
Weeks	1	2	3	4	5	6	7	8
Sessions/week	2	2	3	3	3	3	3	3
Sets	3	4	4	3	4	5	7	7
Repetitions	8	8	8	8	12	12	15	19
Rest between sets (s)	60	60	60	60	60	60	90	90
External load (time under tension)/week (s/week)	474	631	710	533	710	888	404	488
Movement velocity (°s^-1^)	30	40	40	40	60	60	100	140

Both external and internal training load of the isokinetic training were monitored. External training load was determined as the physical work performed during one training session which was measured using the isokinetic dynamometer. Internal training load was determined as the athletes’ rating of perceived exertion (RPE) during the respective sessions. Session RPEs were examined 10 min after each training and testing session. Thereafter, external load was multiplied by internal training session load which resulted in absolute training load (McFarland and Bird, 2014). For the hypertrophy training block, the absolute training loads of all sessions in 1 week were aggregated which resulted in a weekly block training load. For the power block, every session was analyzed as single session and not aggregated in the form of a microcycle.

### Statistical Analyses

Descriptive data are presented as group mean values and standard deviations (SD). Normal distribution was examined using the Shapiro–Wilk test. One-way ANOVA using the factor “time” was conducted to analyze pre-to-post changes in the intervention group. The significance level was set at *p* < 0.05. Additionally, effect sizes (Cohen’s *d*) were calculated using eta-squared. Effect sizes indicate whether a statistically significant difference is a difference of practical concern. In accordance with Cohen (Cohen, 1992), effect sizes were classified as small (0.2 ≤ *d* < 0.49), medium (0.5 ≤*d* < 0.79), and large (*d* ≥ 0.8). Additionally, associations between isokinetic trunk rotator torque, trunk muscle endurance, and canoe-specific performance (i.e., peak paddle force) were assessed using the Pearson product-moment correlation coefficient (*r*-value). Based on the recommendations of Hopkins et al. (2009), values of 0 ≤*r* ≤ 0.69 indicate small, 0.70 ≤*r* ≤ 0.89 medium, and *r* ≥ 0.90 large sized correlations. Statistical analyses were performed using IBM Statistical Package for Social Sciences (SPSS Version 25, SPSS Inc., Chicago, IL, United States).

## Results

### Isokinetic Training

**FIGURE 2:**
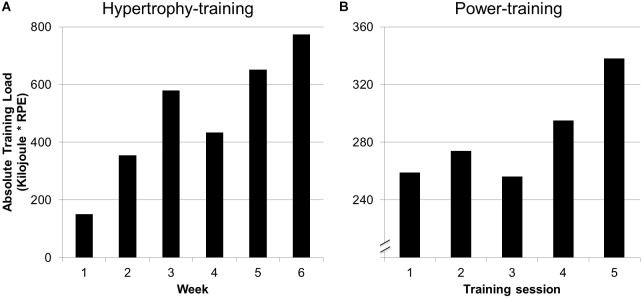
Exemplified documentation of the aggregated absolute training load for every week during the **(A)** hypertrophy block (weeks 1–6) and **(B)** power block (weeks 7–8) of one athlete. The external load (physical work in kilojoule) was multiplied by the internal session load (session rate of perceived exertion) which resulted in absolute training load.

Athletes’ completed the training program with an adherence rate of 88%. Minor adverse health events (colds) were responsible for absence during training. No test or isokinetic training-related injuries occurred over the course of the 8-week training period. Figure 2 shows an example of the absolute training load for the hypertrophy and power training block.

### Body Composition and Muscular Fitness

**FIGURE 3:**
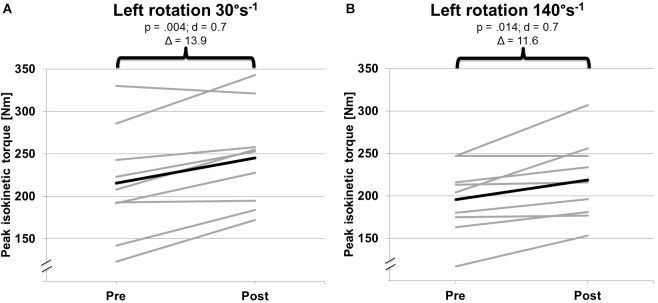
Pre-to-post changes of individual (gray) and mean (black) isokinetic torque in left isokinetic rotation at **(A)** 30°s^-1^ and **(B)** 140°s^-1^ in concentric mode.

**FIGURE 4:**
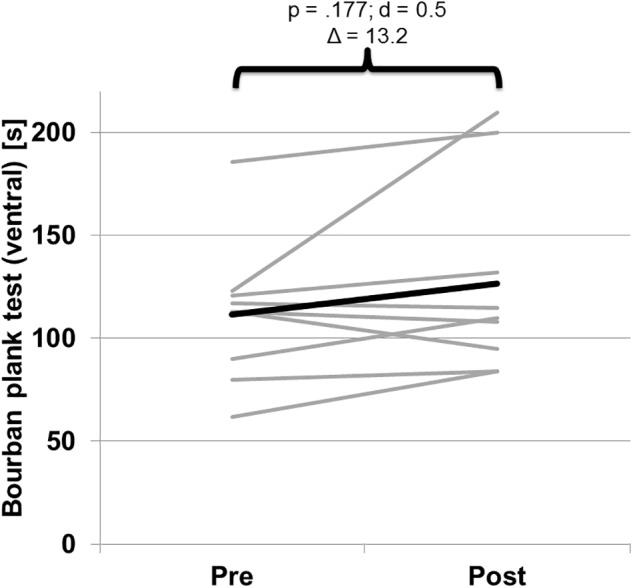
Pre-to-post changes of individual (gray) and mean (black) performance data during the trunk muscle endurance test (Bourban ventral).

No significant pre-to-post changes (*p* ≥ 0.148; *d* ≤ 0.1) were found for any parameter of body composition r (e.g., muscle mass, body fat) (Table 3). Of note, significant pre-to-post increases were detected for peak isokinetic torque during concentric mode of the trunk rotators at 30°s^-1^ and 140°s^-1^ left side but not right side (Table 3). There were no significant differences between left and right rotation in either the pre- or the post-test. Figure 3 shows the individual and mean torque data (concentric) for left rotation. No significant pre-to-post changes were found for peak isokinetic torque (eccentric) of the trunk rotators at 30°s^-1^ and 90°s^-1^ and for the plank test (Figure 4).

**Table 3:** Effects of isokinetic training on body composition, isokinetic torque and trunk muscle endurance in world-class canoeists.

		Pre	Post	*p*-Value (*d*)
*Body composition (n = 6)*				
Body mass (kg)		82.9 ± 9.3	82.6 ± 8.1	0.757 (0.05)
Skeletal muscle mass (kg)		43.6 ± 6.5	43.4 ± 6.2	0.148 (0.03)
Body fat (%)		8.8 ± 4.6	8.7 ± 5.3	0.489 (0.05)
Segmental lean mass of the trunk (kg)		34.0 ± 4.5	33.8 ± 4.1	0.170 (0.07)
Segmental lean mass of the arm	Left	4.7 ± 0.9	4.6 ± 0.8	0.183 (0.06)
	Right	4.7 ± 0.7	4.7 ± 0.7	0.267 (0.02)
Segmental lean mass of the leg	Left	11.2 ± 2.1	11.4 ± 2.2	0.478 (0.12)
	Right	11.3 ± 2.0	11.4 ± 2.2	0.374 (0.11)
*Peak isokinetic trunk rotator torque (n = 9)*			
Peak concentric torque at 30°s^-1^ (Nm)	Left	216 ± 65	245 ± 59	0.004^∗^ (0.68)
	Right	216 ± 66	232 ± 55	0.047^∗^ (0.36)
Peak eccentric torque at 30°/s^-1^ (Nm)	Left	237 ± 72	250 ± 56	0.251 (0.28)
	Right	248 ± 75	248 ± 53	1.000 (0.00)
Peak concentric torque at 140°s^-1^ (Nm)	Left	196 ± 42	219 ± 48	0.014^∗^ (0.72)
	Right	220 ± 52	230 ± 46	0.149 (0.27)
Peak eccentric torque at 90°s^-1^ (Nm)	Left	271 ± 66	284 ± 52	0.260 (0.32)
	Right	256 ± 66	266 ± 62	0.164 (0.22)
*Trunk muscle endurance (n = 9)*				
Bourban plank test (ventral) (s)		112 ± 35	126 ± 47	0.177 (0.50)

*Values are mean and standard deviations. The significance level was set at α < 0.05. Effect sizes were classified according to Cohen. ^∗^Significant pre-to-post difference.*

### Canoe-Specific Performance

**FIGURE 5:**
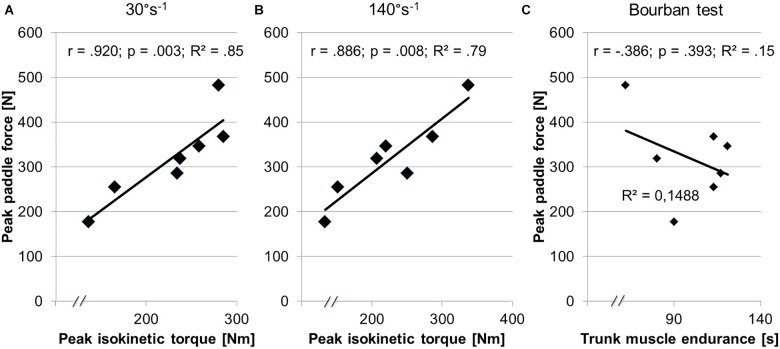
This figure illustrates results from the correlation analysis between canoe-specific performance (i.e., peak paddle force) and peak isokinetic trunk rotator torque during concentric mode at **(A)** 30°s^-1^ and **(B)** 140°s^-1^ and **(C)** the trunk muscle endurance (Bourban) test.

Significant medium-to-high correlations were found between isokinetic trunk rotator torque at (30°s^-1^ and 140°s^-1^) and peak paddle force at 3.4 ms^-1^ during the canoe-specific test in the flume (30°s^-1^: *r* = 0.920; *p* = 0.003; 140°s^-1^: *r* = 0.886; *p* = 0.008). Figure 5 illustrates correlation coefficients between peak paddle force and peak trunk rotator torque at slow and fast movement velocities. The correlational analysis revealed a non-significant and small negative correlation between peak paddle force at 3.4 ms^-1^ boat velocity and performance during the plank (Bourban) test (*r* = -0.386; *p* = 0.393).

## Discussion

This is the first study to examine the effects of additional isokinetic strength training in combination with canoe-specific and athletic training on peak torque of the trunk rotator muscles, trunk muscle endurance and body composition in world-class canoe sprinters.

After 8 weeks of isokinetic trunk rotator training, increases were observed in peak isokinetic torque during slow movement velocity (30°s^-1^) and also fast movement velocities (140°s^-1^). All but one athlete showed improved peak torque (Figure 3).

Our results suggest training-induced improvements in peak torque of the trunk rotator muscles following a combination of canoe-specific training, athletic training and isokinetic training in world-class canoe sprinters. However, the isolated (single-mode) effects of individual training components (canoe vs. athletic vs. isokinetic training) could not be determined due to limitation when conducting research with elite athletes. Additionally, findings from the correlation analyses indicate that isokinetic training may have an impact on canoe-specific performance.

In this study, maximum peak torque during concentric contraction increased over the course of the 8-week training program. Given that there is no study available in the literature that examined the effects of isokinetic trunk rotator training on trunk muscle strength, -mass, and -endurance, we have to relate our findings to studies that conducted isokinetic training for lower and upper limb muscles in different cohorts.

For instance, Housh et al. (1992) examined the effects of an 8-week isokinetic training for the elbow and knee flexors and extensors with three sessions per week on peak torque values of elbow/knee flexors and extensors at 120°s^-1^ in 25-year-old untrained men. The authors observed significant increases in peak torque of the knee/elbow flexors and extensors ranging between 11.2 and 36.0%. In another study, Akima et al. (1999) investigated the effects of a short-term isokinetic knee extensor training at 120°s^-1^ with nine training sessions on peak knee extensor torque at six different angular velocities ranging from 60 to 300°s^-1^ in 24-year-old untrained men. Training resulted in significant increases in peak torque of the knee extensors for angular velocities ranging from 60 to 240°s^-1^ but not for 300°s^-1^. Similar results were reported from Lesmes et al. (1978) who conducted a 7-week isokinetic knee extensor and flexor training at an angular velocity of 180°s^-1^ with four sessions per week in healthy untrained 24-year-old men. Before and after the intervention, peak knee flexor/extensor torque values were obtained at 60, 120, 180, 240, and 300°s^-1^. The authors observed significant increases in peak torque output during 60, 120, and 180°s^-1^ but not for 240 and 300°s^-1^. Finally, Murray et al. (2007) examined the effects of two different angular velocities (60 or 400°s^-1^) during a short-term (eight training sessions) isokinetic training program for the knee extensors and flexors in healthy untrained 24-year-old men and women. In contrast to the previously cited studies, no significant changes were found for peak torque of the knee flexors and extensors during all movement velocities for both training groups (60 or 400°s^-1^) compared with a passive control group.

As a limitation of these studies (Lesmes et al., 1978; Housh et al., 1992; Akima et al., 1999), it has to be noted that no control groups were included in the respective study designs. In agreement with our study, (Lesmes et al., 1978; Housh et al., 1992; Akima et al., 1999) these authors found that isokinetic training increased peak torque at different movement velocities. The results highlighted specific adaptations along the force/torque-velocity spectrum that are in accordance with the principle of training specificity. For this purpose, we decided to increase angular velocity over the 8-week training period to allow adaptations along the force/torque-velocity spectrum. Given that no studies are available in the literature that examined the effects of isokinetic trunk rotator training on trunk muscle strength, -mass, and -endurance, the applied angular velocities were chosen based on piloting work with the included athletes.

In the current study, we were not able to observe increases in eccentric peak torque at either slow or fast movement velocities. However, seven participants showed higher eccentric peak torque at post-test, suggesting that eccentric strength was enhanced in some but not all athletes. Even though some individuals showed increases in eccentric peak torque, this did not result in significant main effects of time for the whole sample. The marginal increase in eccentric peak torque could be attributed to the fact that eccentric training was no longer performed during the final 2-week power block. During the power block also the athletics training (e.g., barbell rowing, barbell bench press) focused on the concentric contraction of the movement. Thus, during the 2-week power block a specific eccentric stimulus was no longer present which may have resulted in detraining of eccentric strength. The absence of a specific training stimulus may explain the unchanged eccentric peak isokinetic torque after the intervention.

Additionally, we were not able to show increases in trunk muscle endurance following isokinetic training. The observed isokinetic training effects on peak torque of the trunk rotators did not translate to improved trunk muscle endurance. On an individual level, we noted that two athletes enhanced their test performance for trunk muscle endurance (Figure 3). Our athletes conducted two blocks of isokinetic training. The first block focused on muscle hypertrophy, the second on muscle power. The intervention resulted in significant pre-to-post increases in peak torque of the trunk rotators at 30°s^-1^ and 140°s^-1^ in concentric mode. This finding is in accordance with the principle of training specificity. However, it also indicates that the applied isokinetic training protocol did not have an impact on trunk muscle endurance. Therefore, if the goal is to improve additionally trunk muscle endurance isokinetic training should be combined with specifically tailored trunk muscle endurance exercises.

Large associations (*r* ≥ 0.886; *p* ≤ 0.008; Figure 5) were found between peak paddle force and peak isokinetic torque in concentric mode. These findings are consistent with the results of Fry and Morton (1991) who reported medium-sized associations between isokinetic torque at 30°s^-1^ (*r* = 0.51-0.66) and at 120°s^-1^ (*r* = 0.46–0.69) and race times over the distances of 500 m up to 42 km in male kayakers who compete in the Western Australian state canoeing championships. In addition, the peak isokinetic torque during the paddle strokes discriminated between kayakers of higher and lower levels (i.e., squad vs. non-squad athlete) in Australian elite canoeists (1991). McKean and Burkett (2014) also examined relationships between the 1-RM pull-up, the 1-RM bench press and 200 m (*r* = 0.63–0.79), 500 m (*r* = 0.66–0.75), and 1,000 m (*r* = 0.59–0.70) time trials in elite kayakers. The present study also showed large relationships between the isokinetic torque of the trunk rotators and the force generated through paddle strokes at 3.4 ms^-1^ in the flume. However, compared to Fry and Morton (1991), the paddle strokes were not simulated but were performed with the individual instrumented paddle in this study.

Based on the findings of Garcia-Pallares et al. (2010), who reported larger gains in paddling speed and paddling power at VO_2max_ after a block periodization of canoe-specific training in elite kayakers in comparison to a traditional periodization, the structure of the isokinetic strength training in this study included a block periodization as well. The desired periodization, which was worked out over the TUT, is reflected (Figure 2) in the product of the actual external (physical work) and internal training load. Based on this ressult, isokinetic training can be planned and periodized using TUT. Additionally, Borges et al. (2014) reported that using session-RPE is a valid method to monitor internal training load, regardless of the RPE scale used.

This study comes with a few limitations that warrant discussion. First, we did not include a passive or an active control group in our study design. However, passive control groups are ethically not justified in elite athletes. Given that the number of world-class athletes in a given sport from one sporting nation is small, we were not able to include an active control group. In this study, sport-specific testing in the flume was only conducted during the baseline tests. Accordingly, it was not possible to deduce effects of isokinetic training on canoe-specific performance. Future studies should examine the long-term effects of isokinetic trunk rotator training in combination with sport-specific and athletic training on canoe-specific performance in contrast to an active control.

## Conclusion

In conclusion, isokinetic trunk rotator training in conjunction with canoe-specific training resulted in increased isokinetic trunk rotator torque (concentric) at slow and fast movement velocities. In addition, a strong relationship was found between peak isokinetic torque and peak paddle force (canoe-specific performance parameter). The current findings suggest that isokinetic training of the trunk rotator muscles may be a promising candidate to improve performance in world-class canoe sprinters. Based on the findings of this study, we recommend to progressively increase angular velocity from 30°s^-1^ to 140°s^-1^ over the course of the training period.

## Author Contributions

All authors listed have made a substantial, direct and intellectual contribution to the work, and approved it for publication.

## Conflict of Interest Statement

The authors declare that the research was conducted in the absence of any commercial or financial relationships that could be construed as a potential conflict of interest.
